# Soil urease inhibition by various plant extracts

**DOI:** 10.1371/journal.pone.0258568

**Published:** 2021-10-14

**Authors:** Muhammad Ajmal Rana, Rashid Mahmood, Sajid Ali

**Affiliations:** 1 Department of Agronomy, University of the Punjab, Lahore, Pakistan; 2 Department of Soil Science, University of the Punjab, Lahore, Pakistan; Bahauddin Zakariya University, PAKISTAN

## Abstract

Urea is the most popular and widely used nitrogenous fertilizer. High soil urease activity rapidly hydrolyses applied urea to ammonia which contributes to soil nitrogen (N) losses and reduces N use efficiency of crop plants. The ammonia losses can be minimized by the inhibition of soil urease activity which has been explored using various potential chemical inhibitors. However, the soil urease activity inhibition potential of plant extracts is rarely explored to date. In the present study, extracts of 35 plant materials were taken and evaluated against jack bean urease. Eleven extracts, showing >50% jack bean urease inhibition, were selected and further investigated in 13 soils collected from various districts of Punjab, Pakistan. Interestingly, except *Capsicum annum*, *Melia azedarach*, *Citrus reticulata* and *Quercus infectoria*, the plant extracts showed urease inhibition activities in soils, the extent of which was lower as compared to that observed in jack bean urease though. Maximum urea hydrolysis inhibition (70%) was noted with *Vachellia nilotica* which was 40% more than that of hydroquinone (50%) followed by that of *Eucalyptus camaldulensis* (24%). The extracts of *V*. *nilotica* and *E*. *camaldulensis* were coated on urea and applied to soil in the next step. At 21^st^ day, 239% and 116% more urea-N was recovered from soil treated with *V*. *nilotica* and *E*. *camaldulensis* extracts coated urea, respectively, as compared to uncoated urea. Conclusively, these results indicated that the coating of *V*. *nilotica* and *E*. *camaldulensis* extracts on urea prills prolonged urea persistence in soil owing to minimum urea hydrolysis, probably, the extracts of *V*. *nilotica* and *E*. *camaldulensis* showed their urease inhibition potential. The results of this study provide a base line for the identification of new soil urease inhibitor compounds from plant materials in future.

## Introduction

Nitrogen (N) is an important macronutrient for plant growth and development primarily due to its role in the biosynthesis of vital molecules such as chlorophyll, amino acids and nucleic acid [1]. Owing to the multiple pathways of nitrogen losses in soils, nitrogen fertilizers are consumed in higher quantities than other fertilizers to ensure sufficient nitrogen provision during the vital growth stages of crop plants, During the year 2018–19, 107 Mt of nitrogen fertilizer consumption was recorded with urea having 55% share alone [2]. Urea, which is a common source of N hydrolyzed to ammonia and carbon dioxide in soil [3]. The uncatalyzed hydrolysis of urea is accelerated by a factor of 8 × 10^7^ due to soil urease activity [4]. This rapid increase in urea hydrolysis is accompanied by a rise in soil pH at the site of hydrolysis [5], and liberation of ammonia [6], which is the highest in low CEC, calcareous [7] and pasture soils [8]. High concentration of free ammonia in soil may damage germinating seeds and young seedlings and if volatilized to the atmosphere it may cause pollution [9]. Furthermore, ammonium accumulation in soil particularly at high pH, may hinder nitrification process at the midway and results in accumulation of toxic levels of nitrites [10].

According to an estimation, excessive soil urease activity in different soils can lost 20–33% applied urea-N on an average which can reach up to 70% [11]. Moreover, ammonia volatilization losses through urea application can reduce nitrogen use efficiency up to 50% in alkaline calcareous soils of arid and semi-arid climate [12].

Studies involving preventing of rapid urea hydrolysis through soil urease inhibition aims to increase nitrogen use efficiency of crop plants [13]. An increase in nitrogen use efficiency means increases in agronomic and economic values of nitrogen fertilizer in terms of increasing crop production and conserving energy as well as resources needed for its manufacturing. Subsequently, minimal nitrogen losses through efficient fertilizer use can minimize environmental pollution [14].

Ureases are nickel-dependent proteinaceous metalloenzymes widely distributed in bacteria, fungi, algae, invertebrates and plants [15]. These are released in soil solution where they persist along with remaining adsorbed to exchange sites [3]. There are various types of soil ureases based on their origin and the compounds inhibiting their activities are also numerous [16]. Among known soil urease inhibitors, N-(n-Butyl)thiophosphoric triamide (NBPT) and hydroquinone are the most popular and potent compounds [17]. Many other compounds like acetohydroxamic acid (AHA) [18], humic acid [19], 1,4-benzoquinone and inorganic metal salts [16] have also been reported for their potential to reduce the activity of soil ureases. Natural plant materials have been extensively investigated to explore their urease inhibition potential but with an aim to get natural remedy against *Helicobacter pylori* infection in human stomach [9,20,21]. However, a few natural materials like *Acacia decurrens*, seed kernel powder of *Azadirachta indica* and bark of *Acacia caven* have also been reported to inhibit ureases in different soils [22].

The use of reported urease inhibitors is limited to certain areas of the world owing to the reasons like cost ineffectiveness, toxicity to crop plants, and variation in effectiveness with soil type, climate and crop management [7]. Therefore, despite a long list of reported inhibitors, need remains in place to search good candidates in the field that are environment friendly, nontoxic to plants, chemically stable, effective in a variety of soils, consistent with urea and cost effective.

This work has proposed a systematic screening for natural soil urease inhibitors starting from the extraction of thirty-five plant materials. Twelve out of these thirty-five extracts have been reported to demonstrate inhibitory activities against jack bean urease, but none has been reported to show inhibition of soil urease activity to date. The study has also optimized the dose of key selected plant extracts for urea coating by studying the impact of coated urea on urea-N stay in soil.

## Materials and methods

### Selection of plant materials and their extraction

Leaves, stem bark, heart wood, fruits, fruit peel and bagasse of thirty-five plant materials were selected based on their ease of availability and cost effectiveness (Table 1). Some of the plant materials included in the study were agricultural wastes.

**Table 1:** Potential of 35 plant extracts to inhibit jack bean urease.

No.	Plants used for extraction			Percent urease inhibition (mean ± SD)
	Botanical name	Common name	Plant part extracted	
**1**	*Nicotiana tabacum*	Tobacco	Leaves	66.14 ± 0.44
**2**	*Quercus infectoria*	Allepo oak	Fruit (galls)	66.60 ± 2.25
**3**	*Moringa oleifera*	Moringa	Leaves	39.38 ± 5.73
**4**	*Brassica rapa*	Turnip	Leaves	49.22 ± 5.43
**5**	*Capsicum annuum*	Bell pepper	Fruit	84.53 ± 0.46
**6**	*Ficus benghalensis*	Banyan	Leaves	24.11 ± 6.41
**7**	*Vachellia nilotica*	Acacia	Leaves	95.27 ± 0.19
**8**	*Eucalyptus camaldulensis*	Eucalyptus	Leaves	92.61 ± 0.44
**9**	*Parthenium hysterophorus*	Carrot grass	Leaves	40.65 ± 2.29
**10**	*Camelia sinensis*	Black Tea	Tea	88.25 ± 0.48
**11**	*Camelia sinensis*	Green Tea	Green tea	81.26 ± 0.89
**12**	*Coffea arabica*	Coffee	Beans	85.79 ± 0.62
**13**	*Azadirachta indica*	Neem	Leaves	28.65 ± 0.82
**14**	*Melia azedarach*	Darek tree	Leaves	89.86 ± 1.33
**15**	*Prunus armeniaca*	Apricot	Leaves	95.16 ± 0.10
**16**	*Psidium guajava*	Guava	Leaves	43.09 ± 1.07
**17**	*Citrus reticulata*	Kinnow	Fruit peel	52.81 ± 4.21
**18**	*Citrus limetta*	Sweet lime	Leaves	38.82 ± 0.70
**19**	*Citrus limon*	Lemon	Leaves	30.83 ± 3.11
**20**	*Citrus sinensis*	Sweet orange	Fruit peel	46.43 ± 2.98
**21**	*Syzygium cumini*	Jambolan	Leaves	33.10 ± 1.88
**22**	*Musa acuminate*	Banana	Fruit peel	0.59 ± 0.39
**23**	*Saccharum officinarum*	Sugarcane	Bagasse	9.25 ± 0.88
**24**	*Allium cepa*	Onion	Leaves	1.05 ± 0.09
**25**	*Mentha arvensis*	Mint	Leaves	34.90 ± 1.06
**26**	*Calotropis gigantean*	Crown flower	Leaves	19.29 ± 0.52
**27**	*Bombax ceiba*	Red cotton tree	Leaves	29.31 ± 0.48
**28**	*Santalum album*	Sandal wood	Heart wood	16.12 ± 0.54
**29**	*Cinnamomum verum*	Cinnamon	Stem bark	30.58 ± 0.68
**30**	*Raphanus sativus* var. longipinnatus	Radish	Leaves	25.20 ± 0.65
**31**	*Olia europaea*	Olive	Leaves	27.12 ± 1.37
**32**	*Tribulus terrestris*	Land Caltrops	Fruit	28.76 ± 0.51
**33**	*Myristica fragrans*	Nutmeg	Fruit	8.41 ± 1.01
**34**	*Brassica oleracea* var. botrytis	Cauliflower	Leaves	21.11 ± 0.97
**35**	*Brassica oleracea* var.capitata	Cabbage	Leaves	20.04 ± 0.50
**Hydroquinone 500 ppm (positive control)**				94.20 ± 1.48
**Acetone 3% (negative control)**				none

Dried and powdered plant materials (10 g) were transferred to conical flasks containing 100 mL of acetone each, and shaked on reciprocal shaker at 200 rpm for 48 hours to obtain extracts. The contents of the flasks were filtered using Whatman filter paper grade 1 and the filtrates were concentrated by solvent evaporation at 70°C to about 3 mL volume that was diluted to 100 mL with distilled water. The extracts were stored maximum up to three days at 4°C if not used immediately in urease inhibition assay.

### Jack bean urease inhibition assay

Jack bean urease inhibition assay was carried out following Nabati et al. [23] with slight modifications. The test reaction mixture was containing 200 μL of 25 mM urea prepared in 100 mM phosphate buffer of pH 6.8, 100 μL of plant extract and 100 μL of urease solution containing 2 mg of jack bean urease (U7752 Sigma-Aldrich) in 1 mL of 100 mM phosphate buffer of pH 6.8. The mixture was incubated at 37°C for 30 minutes followed by dilution to 1 mL by adding 600 μL of distilled water. The blank reaction mixture contained 100 μL of distilled water instead of plant extracts. Hydroquinone (500 ppm) and acetone (3%) solutions were used as positive and negative controls, respectively. All assays were replicated thrice.

After 30 minutes of incubation, ammonia nitrogen (NH_3_-N) in the assay mixtures was determined by following the procedure of Nelson [24] with slight modification. In brief, 0.5 mL of aliquot was diluted to 5 mL with distilled water in a test tube followed by the addition of 0.25 mL of 6% EDTA, 1 mL of sodium salicylate—sodium nitroprusside reagent (7.82 g sodium salicylate and 0.125 g sodium nitroprusside dissolved in distilled water to make total volume 100 mL) and 0.5 mL of freshly prepared buffered hypochlorite (2.96 g sodium hydroxide, 9.96 g sodium hydrogen phosphate heptahydrate and 10 mL sodium hypochlorite dissolved in distilled water to make a total volume of 100 mL). The mixture was incubated at 37°C in water bath for 30 minutes. Green color intensity was measured at 667 nm by using ultraviolet-visible spectrophotometer and NH_3_-N in the aliquot was estimated from the curve of NH_3_-N standards. Urease inhibition by a plant extract was calculated by the following formula [20].


$$I(\%)=\left(1-\frac{T}{C}\right)\times 100$$

Where, I (%) is the percent urease inhibition, T and C are NH_3_-N concentrations in the test and blank reaction mixtures, respectively.

### Soil sampling and analysis

Thirteen soil samples were collected from Lahore, Sheikhupura, Gujranwala, Jhang, Narowal, Okara, Khanewal, Sahiwal and Multan districts of Punjab, Pakistan. These soils have already been classified to subgroup level by Soil Survey of Punjab, Pakistan. Textures of the soils were estimated by hydrometer method [25]. Soil organic matter was estimated by Walkley and Black method [26]. Cation exchange capacity of the soils was estimated by loading sodium ions to the exchange sites followed by unloading and flame photometric estimation [27]. For Na, K, Ca and Mg determinations, soils were extracted with ammonium acetate solution and concentrations of these ions in extracts were determined by atomic absorption spectrophotometry [28]. Soil urease activity was estimated by incubating soil with urea for 2 hours at 37°C followed by ammonium determination through modified Berthelot reaction [29] (Table 2).

**Table 2:** Characteristics of thirteen soils used in urease inhibition assay using plant extracts.

Soil No.	Sub-group	Sand	Silt	Clay	Organic matter	Cation exchange capacity	pH	K	Na	Ca	Mg	Urease activity (μg N g^-1^ 2h^-1^)
		%				meq 100 g^-1^		mg g^-1^				
**1**	Ustic Torrrifluvents	21.0	58.5	20.5	0.58	6.3	7.8	0.09	0.06	1.76	0.54	100
**2**	Ustic Torrrifluvents	33.5	51.0	15.5	0.61	14.9	8.3	0.10	0.11	3.03	0.93	135
**3**	Fluventic Haplocambids	16.0	61.0	23.0	0.78	8.5	8.0	0.24	0.40	1.43	0.47	191
**4**	Typic Hapustalfs	33.5	46.0	20.5	0.70	7.7	8.2	0.14	0.12	2.67	0.84	141
**5**	Typic Haplosalids	45.0	40.0	15.0	0.84	13.3	8.7	0.07	0.06	2.41	0.75	175
**6**	Ustic Haplocambids	33.5	43.5	23.0	0.87	9.7	8.3	0.16	0.17	2.28	0.69	139
**7**	Typic Torrrifluvents	56.0	26.0	18.0	0.67	10.9	7.7	0.14	0.26	5.73	0.73	182
**8**	TypicTorripsamments	7.9	80.0	12.1	0.20	5.6	8.7	0.08	0.09	1.30	0.41	36
**9**	Typic Calciargids	7.5	67.0	25.5	0.90	11.3	8.6	0.17	0.17	2.67	1.12	161
**10**	Fluventic Eutrudepts	28.5	47.5	24.0	0.55	10.6	8.3	0.27	0.20	5.01	1.56	160
**11**	Ustic Calciargids	31.5	52.0	16.5	0.61	10.0	9.4	0.24	0.39	5.25	1.55	138
**12**	Typic Haplocambids	14.5	70.5	15.0	0.93	10.5	8.1	0.11	0.13	3.74	1.12	130
**13**	Fluventic Haplustepts	20.0	50.0	30.0	1.02	14.7	8.4	0.18	0.20	5.51	1.51	173

### Urea hydrolysis inhibition in soils

The eleven plant extracts which showed more than 50% inhibition of jack bean urease were further investigated for urea hydrolysis inhibition in the thirteen soils. These soils belonged to four soil orders i.e., aridisols, alfisols, entisols and inceptisols and twelve subgroups. The soils were varied in organic matter from 0.20 to 1.02%, pH from 7.7 to 9.4, CEC from 5.6 to 14.9 meq 100 g^-1^ and urease activity from 36 to 673 μg urea-N hydrolyzed g^-1^ 2h^-1^. Ammonium acetate extractable K, Na, Ca, and Mg were varied from 0.07 to 0.27, 0.06 to 0.39, 1.30 to 5.51 and 0.41 to 1.56 mg g^-1^, respectively (Table 2).

The reaction mixture comprised of 1 mL of plant extract, 1 mL of 25 mM urea solution and 1 g of soil. After 2 hours of incubation at 37°C, assay mixture was diluted by adding 5 mL of distilled water followed by centrifugation at 6000 rpm for 10 minutes. The supernatant was used for ammonia determination as described earlier [29]. Each soil-extract assay was replicated thrice and an assay mixture with a soil but without an extract was considered as blank for that soil. Urea hydrolysis inhibition was determined by the same formula as discussed in case of jack bean urease inhibition assay previously. Hydroquinone solution (500 ppm) was used as reference urea hydrolysis inhibitor in soils.

### Preparation of plant extracts-coated urea

In soil assays, maximum urea hydrolysis inhibition was noted with extract of *Vachellia nilotica* followed by that of *Eucalyptus camaldulensis*. The extracts of these plants were further studied by coating them on urea prills after extracting their 10, 20, 50 and 100 g of dried and powdered leaves with double the amount of acetone separately following the same procedure as discussed earlier. The coating of urea was done by pouring a concentrated extract (2 mL) over 100 g of urea prills rotating in a rotary mixing container till uniform application of the extract over the surface of the prills. The coated urea prills were then removed from the container and dried in shade.

### Effectiveness of plant extracts-coated urea for urea-N recovery from soil

**Fig 1:**
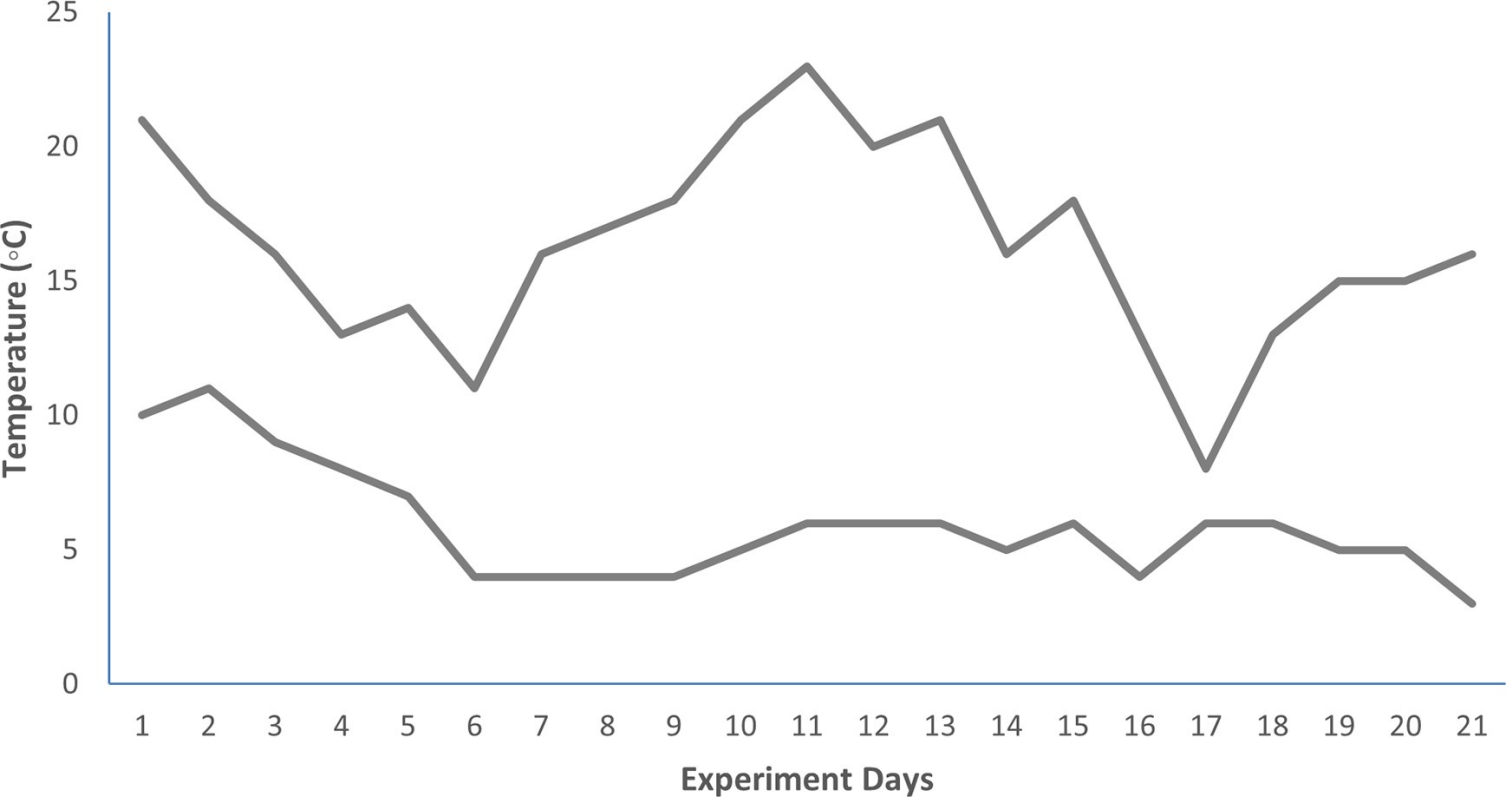
Daily minimum and maximum air temperatures during pot trial of extract coated urea fertilizer.

Surface field soil of 0–15 cm depth (soil no. 1 in Table 2) was collected, sieved, and filled to polythene lined earthen pots to a capacity of 600 g soil per pot. The experiment was conducted in a completely randomized design (CRD) with and type of plant extract and dose of plant extract coating urea as two experimental factors. Urea coated with extracts of 0, 10, 20, 50 and 100 g dried leaves of *V*. *nilotica* and *E*. *camaldulensis* was applied at 225 mg urea-N kg^-1^ soil in triplicate. The pot soil was irrigated with tap water and moisture level was maintained to field capacity. The pots were placed in an open corridor at natural temperature for 21 days. Variations in daily minimum and maximum temperatures are presented in Fig 1. After 7, 14 and 21 days of incubation, each pot soil was sampled for residual urea determination.

Urea from the soil (2 g dry matter) was extracted with 2 M KCl-PMA (KCl-phenyl mercuric acetate) solution. A known volume of the extract was then incubated at 85°C for 45 minutes with a 5:2:100 mixture of diacetyle monoxyme (DAM) solution, thiosemicarbazide (TSC) solution and acid reagent in test tubes. After incubation the test tubes were immediately cooled in running tap water and red color intensity of the contents was measured using spectrophotometer at 527 nm [30].

### Statistical analysis

The data of all the experiments were subjected to analysis of variance and means were compared by Tukey’s HSD test. All the statistical analyses were performed by Statistix-8.1 software.

## Results

Maximum inhibition of jack bean urease was noted with *V*. *nilotica* and *Prunus armeniaca*, (both 95%), which was comparable to that of hydroquinone (94%), followed by that of *E*. *camaldulensis* (92%) and *Melia azedarach* (89%) (Table 1). The extracts of *Nicotiana tabacum*, *Quercus infectoria*, *Capsicum annuum*, *Camellia sinensis* (green tea), *C*. *sinensis* (black tea), *Coffea Arabica*, *Citrus reticulata* also showed more than 50% inhibition of jack bean urease (Table 1).

The eleven extracts which inhibited jack bean urease more than 50%, were further investigated in thirteen soils characterized in Table 2. The extracts of *C*. *annum*, *M*. *azedarach* and *C*. *reticulata*, increased urea hydrolysis instead of inhibition in all thirteen soils. *Quercus infectoria* extract increased urea hydrolysis in seven soils and inhibited it slightly up to 14% in other six soils. The data of these four extracts which stimulated urea hydrolysis in most of the soils were neither subjected to analysis of variance nor presented in this manuscript. Other seven extracts inhibited soil urease without showing any variation in their inhibition potential among the soils. *Vachellia nilotica* extract demonstrated maximum inhibition (70% on an average) in all thirteen soils which was 40% more than that showed by hydroquinone (49.5%). The extracts of *E*. *camaldulensis*, *C*. *sinensis* (green) and *P*. *armeniaca* followed *V*. *nilotica* and showed urease hydrolysis inhibitions of 24%, 22% and 20%, respectively, on average. *Nicotiana tabacum* showed 3.5% inhibition on average basis (Table 3).

**Table 3:** Urea hydrolysis inhibition in thirteen soils estimated in an In-vitro assay involving an incubation of soil with urea at 37°C for two hours.

Soil	*Nicotiana tabacum*	*Vachellia nilotica*	*Eucalyptus camaldulensis*	*Camelia sinensis (black)*	*Camelia sinensis (green)*	*Coffea arabica*	*Prunus armeniaca*	Hydroquinone
**1**	3.0 t-v	70.7 a-d	23.9 j-n	15.6 k-v	24.2 j-n	2.4 v	23.6 j-o	51.0 e-h
**2**	3. t-v	67.0 a-f	24.7 j-n	20.4 j-v	21.9 j-s	3.6 s-v	21.7 j-t	51.7 e-h
**3**	4.0 r-v	82.1 a	22.7 j-r	38.5 h-j	23.2 j-o	6.3 n-v	24.4 j-n	49.0 f-h
**4**	4.0 r-v	66.6 a-f	24.4 j-n	11.0 k-v	21.5 j-t	7.4 m-v	21.4 j-t	54.0 b-h
**5**	2.6 uv	78.8 a	24.3 j-n	5.0 o-v	25.6 j-m	4.1 p-v	20.4 j-v	47.3 gh
**6**	3.9 r-v	68.3 a-e	24.2 j-n	8.9 l-v	17.8 k-v	6.0 n-v	14.9 k-v	47.0 gh
**7**	3.3 s-v	68.8 a-e	28.1 i-k	20.5 j-v	22.8 j-q	2.0 v	20.5 j-v	46.0 g-i
**8**	4.4 p-v	67.2 a-f	20.3 j-v	7.6 m-v	20.4 j-v	3.4 s-v	16.6 k-v	52.7 c-h
**9**	3.3 s-v	69.3 a-e	26.5 j-l	10.3 k-v	20.2 j-v	7.9 l-v	17.9 k-v	51.3 e-h
**10**	3.0 t-v	64.1 a-g	23.0 j-p	7.9 l-v	18.9 k-v	6.1 n-v	13.3 k-v	47.0 gh
**11**	3.0 t-v	72.7 ab	28.0 i-k	19.9 j-v	23.6 j-o	13.3 k-v	22.1 j-s	47.0 gh
**12**	3.9 r-v	71.4 a-c	23.8 j-o	19.4 k-v	24.4 j-n	6.7 n-v	21.3 j-u	52.0 d-h
**13**	4.0 r-v	67.2 a-f	23.6 j-o	19.1 k-v	21.4 j-t	8.4 l-v	22.1 j-s	47.3 gh
**Mean**	3.5 F	70.4 A	24.4 C	15.7 E	22.0 CD	6.0 F	20.0 D	49.5 B

*Means sharing common letter(s), lower case in the whole table except last row and upper case in the last row, do not differ significantly at p ≤ 0.05.

Having identified their potential of urease activity inhibition with the extracts of *V*. *nilotica* and *E*. *camaldulensis*, they were coated on urea prills and incubated with soil-1 for 21 days. The impact of extract coated urea on urea-N recovery at 7^th^, 14^th^ and 21^st^ day of incubation (DOI) is presented in Table 4. In comparison to uncoated urea, application of *V*. *nilotica* extract coated urea significantly increased urea recovery at all three sampling times. Recovery of urea, coated with extract of 10 g dry leaves per 100 g urea, was 16, 27 and 168% more than that of uncoated urea at 7^th^, 14^th^ and 21^st^ DOI, respectively. Coating with extract of 20 g *V*. *nilotica* leaves increased urea recovery to 23% at 7^th^, 45% at 14^th^ and 239% at 21^st^ DOI. Coating of extracts taken from 50 and 100 g leaves did not further increase urea recovery in comparison to extract of 20 g leaves (Table 4).

**Table 4:** Percent recovery of urea-N from soil incubated with urea coated with leaf extracts of *Vachellia nilotica* and *Eucalyptus camaldulensis*.

Extract coated	Extract dose (g dry leaves per 100 g urea)δ	Urea-N recovery (%)		
		7^th^ day	14^th^ day	21^st^ day
**Control (uncoated urea)**		79.9 d*	26.9 d	6.1 d
***V*. *nilotica***	10	93.1 b	34.3 b	16.4 bc
	20	98.5 a	39.2 a	20.7 ab
	50	98.6 a	39.8 a	21.7 a
	100	98.7 a	39.8 a	21.6 a
***E*. *camaldulensis***	10	87.6 c	29.0 cd	13.2 c
	20	88.0 c	30.7 c	14.9 c
	50	90.6 bc	31.3 c	16.3 bc
	100	91.0 bc	31.0 c	16.1 bc
**Hydroquinone**		82.1 d	31.2 c	21.5 a
**HSD (p ≤ 0.05)**		5.06	2.58	5.18

δPlant leaves were dried and extracted with double amount of acetone, the volume of which was reduced to 2 mL by vacuum evaporation and coated to 100 g urea.

*Means sharing common letter(s) in a column do not differ significantly as compared by Tukey’s HSD test at p ≤ 0.05.

Difference in urea-N recovery between uncoated and coated urea with extract of 10 g leaves of *E*. *camaldulensis* was about 9% at 7^th^ DOI which increased to 116% at 21^st^ DOI. Increasing weight of extracted leaves from 10 g up to 100 g did not significantly (p ≤ 0.05) increased urea recovery at any of the sampling times (Table 4).

## Discussion

The extraction of plant materials, to study their urease inhibition potential, is carried out mostly through water, methanol or acetone as extracting solvents [23,31]. In our study we preferred acetone, as an extracting solvent, over water or methanol, due to several reasons. Firstly, in parallel extraction of a plant materials with water, methanol and acetone, acetone extracts showed more urease inhibition as compared to the extracts from other two solvents [31]. Secondly, in our preliminary trials, the urease activity inhibition with acetone extracted plant materials was more consistent with second and/or third time sampling of plant materials than the extracts taken through water and methanol. It could be due to the possible extraction of plant ureases along with inhibitory compounds from plant materials extracted with water and methanol as plants varied in urease concentration that depends upon plant growth factors [32,33]. Another possible reason of consistent urease activity inhibition potential of acetone extracted plant materials could be the insolubility of ureases and other proteins in acetone which ensures the extraction of inhibitory compounds only [34].

Twelve out of 35 investigated plant materials, in this study, are already reported to have inhibition potential against jack bean urease. In our study, most of these materials did not perform the same way as reported in literature. According to literature 50% methanolic extracts of *Q*. *infectoria*, *Saccharum officinarum*, *Allium cepa*, *Mentha arvensis*, *Santalum album*, *Cinnamomum verum*, *Olia europaea* and *Myristica fragrans* inhibited activity of jack bean urease by 98%, 35%, 53%, 93%, 59%, 84%, 72%, and 78% respectively [23]. However, in our study the inhibitions due to these materials were far below their reported percentages (Table 1). Nonetheless, the extracts of *N*. *tabacum*, *V*. *nilotica*, *C*. *sinensis* and *Psidium guajava* showed jack bean urease inhibition comparable to or greater than that reported in literature [23,31]. These contradictions might be due to owing to their spaciotemporal distribution and geographical location [35], whereby different varieties of a same plant may have a different profile of metabolites [36]. Other factors like plant growth factors [37] stage of a plant at the time of material collection [38], method of extraction, quantity of plant material extracted, and extract concentration used in assays may also affect the chemical composition of extracts [23,31].

In comparison to jack bean urease, reduction in inhibition potential or failure of some plant extracts in soils was, perhaps, due to the fact that soil ureases being a mixture of plant, fungal and bacterial ureases are different from pure jack bean urease. As plant and fungal ureases are monotrimers or hexamers of about 90 kDa subunits, while bacterial ureases are multimers of two or three subunits complexes [39,40]. Furthermore, apart from their presence in soil solution, soil ureases are also found on exchange sites which might have contributed in urea hydrolysis reaction [3]. Additionally, soil contains several organic and inorganic molecules which could have disturbed the overall electrolyte concentration of the assay solution mixture and interfered with urease inhibition process in our study [41].

The screening through jack bean urease, in comparison to soil, is easier, free from interferences and gives precise results [42]. These factors support the use of jack bean urease during initial large-scale screening of new compounds and extracts for their urease inhibition potential, to identify the best performers to be investigated further in soils. In general, it can be inferred from the better performance of all eleven extracts in jack bean urease assay than in soil assay that a compound/extract which is not inhibitory to jack bean urease will not inhibit soil ureases.

## Conclusion

The results of the experiments provide a base line for the identification and extraction of new urease inhibitor compounds from the effective plant extracts. Leaves of *V*. *nilotica* and *E*. *camaldulensis* can be extracted with acetone to coat urea, particularly in the regions where these species are in abundance. These coatings can delay urea hydrolysis minimum up to three weeks to increase urea-N efficiency in alkaline soils. Further investigations regarding any possible allelopathic effects of extracts coatings on different crop plants are needed in future.

## References

[1] Marschner H. Mineral nutrition of higher plants. Academic press; 2011.

[2] Heffer P, Prud’homme M. Fertilizer Outlook 2017–2021. Proceedings of the 85th IFA Annual Conference, Marrakech, Morocco. 2017. pp. 22–24.

[3] Cantarella H, Otto R, Rodrigues J, Gomes A, Silva DB. Agronomic efficiency of NBPT as a urease inhibitor: A review. J Adv Res. 2018;13: 19–27. doi: 10.1016/j.jare.2018.05.008 30094079 PMC6077139

[4] Callahan BP, Yuan Y, Wolfenden R. The burden borne by urease. J Am Chem Soc. 2005;127: 10828–10829. doi: 10.1021/ja0525399 16076178

[5] Chung H, Kim SH, Nam K. Inhibition of urea hydrolysis by free Cu concentration of soil solution in microbially induced calcium carbonate precipitation. Sci Total Environ. 2020;740: 140194. doi: 10.1016/j.scitotenv.2020.140194 32563888

[6] Sigurdarson JJ, Svane S, Karring H. The molecular processes of urea hydrolysis in relation to ammonia emissions from agriculture. Rev Environ Sci Bio/Technology. 2018;17: 241–258. doi: 10.1007/s11157-018-9466-1

[7] Sunderlage B, Cook RL. Soil Property and Fertilizer Additive Effects on Ammonia Volatilization from Urea. Soil Sci Soc Am J. 2018;82: 253–259. doi: 10.2136/sssaj2017.05.0151

[8] Smith AP, Johnson IR, Schwenke G, Lam SK, Suter HC, Eckard RJ. Predicting ammonia volatilization from fertilized pastures used for grazing. Agric For Meteorol. 2020;287: 107952. doi: 10.1016/j.agrformet.2020.107952.

[9] Modolo L V., de Souza AX, Horta LP, Araujo DP, de Fátima Â. An overview on the potential of natural products as ureases inhibitors: A review. J Adv Res. 2015;6: 35–44. doi: 10.1016/j.jare.2014.09.001 25685542 PMC4293669

[10] Breuillin-Sessoms F, Venterea RT, Sadowsky MJ, Coulter JA, Clough TJ, Wang P. Nitrification gene ratio and free ammonia explain nitrite and nitrous oxide production in urea-amended soils. Soil Biol Biochem. 2017;111: 143–153.

[11] Kiss S, Simihaian M. Improving efficiency of urea fertilizers by inhibition of soil urease activity. Springer Science & Business Media; 2002.

[12] Francisco SS, Urrutia O, Martin V, Peristeropoulos A, Garcia-mina JM. Efficiency of urease and nitrification inhibitors in reducing ammonia volatilization from diverse nitrogen fertilizers applied to different soil types and wheat straw mulching. 2011;91: 1569–1575. doi: 10.1002/jsfa.4349 21656770

[13] Allende-Montalbán R, Martín-Lammerding D, Delgado M del M, Porcel MA, Gabriel JL. Urease Inhibitors Effects on the Nitrogen Use Efficiency in a Maize–Wheat Rotation with or without Water Deficit. Agriculture. 2021;11: 684. doi: 10.3390/agriculture11070684

[14] Park SH, Lee BR, Kim TH. Urease and nitrification inhibitors with pig slurry effects on ammonia and nitrous oxide emissions, nitrate leaching, and nitrogen use efficiency in perennial ryegrass sward. Asian-Australasian J Anim Sci. 2021. doi: 10.5713/ab.21.0046 PMC8563229 33902171

[15] Carlini CR, Ligabue-braun R. Ureases as multifunctional toxic proteins: A review. Toxicon. 2016;110: 90–109. doi: 10.1016/j.toxicon.2015.11.020 26690979

[16] Modolo L V, Da-Silva CJ, Brandão DS, Chaves IS. A minireview on what we have learned about urease inhibitors of agricultural interest since mid-2000s. J Adv Res. 2018;13: 29–37. doi: 10.1016/j.jare.2018.04.001 30094080 PMC6077229

[17] Li W, Xiao Q, Hu C, Liu B, Sun R. A comparison of the efficiency of different urease inhibitors and their effects on soil prokaryotic community in a short-term incubation experiment. Geoderma. 2019;354: 113877. doi: 10.1016/j.geoderma.2019.07.035

[18] Azizian H, Esmailnejad A, Fathi Vavsari V, Mahernia S, Amanlou M, Balalaie S. Pantoprazole derivatives: Synthesis, urease inhibition assay and in silico molecular modeling studies. ChemistrySelect. 2020;5: 4580–4587. doi: 10.1002/slct.202000578

[19] Liu X, Zhang M, Li Z, Zhang C, Wan C, Zhang Y, et al. Inhibition of urease activity by humic acid extracted from sludge fermentation liquid. Bioresour Technol. 2019;290: 121767. doi: 10.1016/j.biortech.2019.121767 31302466

[20] Mahernia S, Bagherzadeh K, Mojab F, Amanlou M. Urease inhibitory activities of some commonly consumed herbal medicines. Iran J Pharm Res. 2015;14: 943–947. Available: http://www.pubmedcentral.nih.gov/articlerender.fcgi?artid=4518124&tool=pmcentrez&rendertype=abstract. 26330884 PMC4518124

[21] Abbas HMK, Huang HX, Huang WJ, Xue SD, Yan SJ, Wu TQ, et al. Evaluation of metabolites and antioxidant activity in pumpkin species. Nat Prod Commun. 2020;15: 1–11. doi: 10.1177/1934578X20920983

[22] Mathialagan R, Mansor N, Al-Khateeb B, Mohamad MH, Shamsuddin MR. Evaluation of allicin as soil urease inhibitor. Procedia Eng. 2017;184: 449–459. doi: 10.1016/j.proeng.2017.04.116

[23] Nabati F, Mojab F, Habibi-rezaei M, Bagherzadeh K, Amanlou M, Yousefi B, et al. Large scale screening of commonly used Iranian traditional medicinal plants against urease activity. 2012;20: 72. doi: 10.1186/2008-2231-20-72 23351780 PMC3556030

[24] Nelson DW. Determination of ammonium in KCl extracts of soils by the salicylate method. Commun Soil Sci Plant Anal. 1983;14: 1051–1062. doi: 10.1080/00103628309367431

[25] Gee GW, Bauder J. W., 1986. Particle-Size Analysis. Methods soil Anal Part. 1986;1: 404–407.

[26] Jha P, Biswas AK, Lakaria BL, Saha R, Singh M, Rao AS. Predicting total organic carbon content of soils from Walkley and Black analysis. Commun Soil Sci Plant Anal. 2014;45: 713–725. doi: 10.1080/00103624.2013.874023

[27] Rhoades JD. Cation exchange capacity. Methods Soil Anal Part 2 Chem Microbiol Prop. 1983;9: 149–157.

[28] Warncke D, Brown JR. Potassium and other basic cations. Recomm Chem soil test Proced North Cent Reg. 1998;1001: 31.

[29] Kandeler E, Gerber H. Short-term assay of soil urease activity using colorimetric determination of ammonium. Biol Fertil Soils. 1988;6: 68–72. doi: 10.1007/BF00257924

[30] Douglas LA, Bremner JM. Extraction and colorimetric determination of urea in soils. Soil Sci Soc Am J. 1970;34: 859–862. doi: 10.2136/sssaj1970.03615995003400060015x

[31] Amin M, Anwar F, Naz F, Mehmood T, Saari N. Anti-Helicobacter pylori and urease inhibition activities of some traditional medicinal plants. Molecules. 2013;18: 2135–2149. doi: 10.3390/molecules18022135 23434867 PMC6270356

[32] Nhung HTN, Loan HTT, Lam TB. Effects of germination time of soybean to the activity of soybean urease enzyme. Bioresearch Commun. 2019;5: 606–609. Available: https://www.bioresearchcommunications.com/index.php/brc/article/view/128/122.

[33] Aguetoni Cambuí C, Gaspar M lia, Mercier H. Detection of urease in the cell wall and membranes from leaf tissues of bromeliad species. Physiol Plant. 2009;136: 86–93. doi: 10.1111/j.1399-3054.2009.01217.x 19508368

[34] Crowell AMJ, Wall MJ, Doucette AA. Maximizing recovery of water-soluble proteins through acetone precipitation. Anal Chim Acta. 2013;796: 48–54. doi: 10.1016/j.aca.2013.08.005 24016582

[35] Swab RM, Regan HM, Matthies D, Becker U, Bruun HH. The role of demography, intra‐species variation, and species distribution models in species’ projections under climate change. Ecography (Cop). 2015;38: 221–230. doi: 10.1111/ecog.00585

[36] Verma N, Shukla S. Impact of various factors responsible for fluctuation in plant secondary metabolites. J Appl Res Med Aromat Plants. 2015;2: 105–113. doi: 10.1016/j.jarmap.2015.09.002

[37] Del-Castillo-Alonso MÁ, Castagna A, Csepregi K, Hideg É, Jakab G, Jansen MAK, et al. Environmental factors correlated with the metabolite profile of Vitis vinifera cv. Pinot Noir berry skins along a European latitudinal gradient. J Agric Food Chem. 2016;64: 8722–8734. doi: 10.1021/acs.jafc.6b03272 27794599

[38] Ryu HW, Yuk HJ, An JH, Kim D-Y, Song H-H, Oh S-R. Comparison of secondary metabolite changes in Camellia sinensis leaves depending on the growth stage. Food Control. 2017;73: 916–921. doi: 10.1016/j.foodcont.2016.10.017

[39] Mobley HL, Island MD, Hausinger RP. Molecular biology of microbial ureases. Microbiol Rev. 1995;59: 451–480. doi: 10.1128/mr.59.3.451-480.1995 7565414 PMC239369

[40] Sirko A, Brodzik R. Plant ureases: roles and regulation. Acta Biochim Pol. 2000;47: 1189–1195. doi: 10.18388/abp.2000_3972 11996109

[41] Amtul Z, Siddiqui RA, Choudhary MI. Chemistry and mechanism of urease inhibition. Curr Med Chem. 2002;9: 1323–1348. doi: 10.2174/0929867023369853 12132990

[42] Kataria R, Khatkar A. Molecular docking of natural phenolic compounds for the screening of urease inhibitors. Curr Pharm Biotechnol. 2019;20: 410–421. doi: 10.2174/1389201020666190409110948 30963969

